# Synchronous Multiple Breast Cancers—Do We Need to Reshape Staging?

**DOI:** 10.3390/medicina56050230

**Published:** 2020-05-11

**Authors:** Minodora Onisâi, Adrian Dumitru, Iuliana Iordan, Cătălin Aliuș, Oana Teodor, Adrian Alexandru, Daniela Gheorghiță, Iulian Antoniac, Adriana Nica, Alexandra-Ana Mihăilescu, Sebastian Grădinaru

**Affiliations:** 1 Faculty of General Medicine, University of Medicine and Pharmacy Carol Davila Bucharest, 050474 Bucharest, Romania; minodorel@yahoo.com (M.O.); dr.adrianalexandru@yahoo.com (A.A.); adriana.nica@suub.ro (A.N.); gradinarusebastian@gmail.com (S.G.); 2 Hematology Department, Emergency University Hospital Bucharest, 050098 Bucharest, Romania; iulia.iordan@yahoo.com; 3 Pathological Anatomy Department, Emergency University Hospital Bucharest, 050098 Bucharest, Romania; dr.adriandumitru@gmail.com; 4 4th Surgery Department, Emergency University Hospital Bucharest, 050098 Bucharest, Romania; alius.catalin@gmail.com; 5 Obstetrics-Gynecology Department, Emergency University Hospital Bucharest, 050098 Bucharest, Romania; teodor.oana.mihaela@gmail.com; 6 Plastic Reconstructive Surgery Department, Emergency University Hospital Bucharest, 050098 Bucharest, Romania; 7 Faculty of Materials Science and Engineering, University Politehnica of Bucharest, 060042 Bucharest, Romania; antoniac.iulian@gmail.com; 8 Academy of Romanian Scientists, 050094 Bucharest, Romania; 9 Intensive Care Unit, Emergency University Hospital Bucharest, 050098 Bucharest, Romania; 10 Intensive Care Unit, Foisor Hospital Bucharest, 030167 Bucharest, Romania; mihailescu87alexandra@gmail.com

**Keywords:** breast cancer, tumors, lobular carcinoma, histopathological characteristics, mismatch

## Abstract

*Background and Objectives:* Current recommendations and treatment regimens in breast cancer are a reflection of its heterogeneity on multiple levels including histological subtypes, grading, molecular profiling, and numerous prognostic indices. Although based on extensive research, current guidelines are not explicit in the case of surgical specimens showing various degrees of mismatch between different parts of the same tumor and even more so between multicentric lesions. Synchronous breast cancer is the ideal prototype for studying inter- and intra-tumoral heterogeneity, therefore we envisaged that a study on patients with multicentric and multifocal lesions could contribute to the reshaping of the staging, prognosis, and treatment of breast malignancies. *Material and Methods:* A prospective observational study was conducted between January 2013 and May 2017 on 235 patients diagnosed with breast cancer (BC) and surgically treated at Emergency University Hospital, Bucharest. Thirty-seven patients had multiple breast tumors and were eligible for assessment of the heterogeneity of their lesions. *Results*: 6 were multicentric and 31 multifocal. The number of foci varied from 2 to 11. We encountered numerous mismatches between the index and the secondary tumors, as follows: 3 cases (8.1%) with histopathological mismatch, 13 (35.1%) with different grades of differentiation, 11 (29.8%) with ER (Estrogen Receptors) status mismatch, 12 (32.4%) with PR (Progesterone Receptors) status mismatch, 8 (21.6%) with molecular phenotype mismatch, and 17 (45.9%) cases with variable Ki-67. After careful analysis of index and secondary tumors, apart from the mismatches reported above, we discovered that the secondary tumors were actually dominant in 5 cases (13.5%), and therefore at least those cases had to be reclassified/restaged, as the supplementary data commanded changes in the therapeutic decision. *Conclusions:* For synchronous breast tumors, the current Tumor-Node-Metastasis (TNM) staging system ignores not only the histopathological and immunohistochemical characteristics of the secondary foci, but also their size. When secondary lesions are more aggressive or their cumulative mass is significantly bigger than that of the index tumor, the treatment plan should be adapted accordingly. We believe that information obtained from examining secondary foci in synchronous breast cancer and assessment of the cumulative tumoral mass should be reflected in the final staging and definitive treatment. The clinical benefit of staging the patients based on the most aggressive tumor and the cumulative tumoral burden rather than according to the biggest single tumor, will avoid under-treatment in cases with multifocal/multicentric BC displaying intertumoral mismatch.

## 1. Introduction

Breast cancer is a condition that displays a high heterogeneity, both inter- and intra-tumoral, therefore multiple subtypes can coexist within a tumor [1]. Synchronous multiple breast malignant tumors include multifocality and multicentricity. Multifocality is described as the simultaneous presence of two or more tumoral foci in one quadrant, while multicentricity is used for tumors developing in distinctive quadrants [2]. The incidence of multiple breast cancer ranges from 10% to 24% of all breast cancers [2,3]. The influence of synchronous tumors on prognosis is highly controversial. The current edition of Tumor-Node-Metastasis (TNM) staging system uses only the dimension of the biggest tumor [4]. Several studies analyzed the importance of multiplicity in prognosis and considered reassessment of classifications in order to include this aspect [5,6,7,8,9,10]. This study aims to evaluate the synchronous multiple breast cancer to determine the importance of the evaluation of multifocality and multicentricity. We also analyzed the necessity of including histopathological and immunohistochemical heterogeneity in the current staging systems, considering that a different classification might change the management.

## 2. Materials and Methods

A descriptive prospective observational study was conducted between January 2013 and May 2017, comprising of 235 patients diagnosed with breast cancer and surgically treated in the departments of General Surgery and Obstetrics-Gynecology at Emergency University Hospital, Bucharest, Romania. We excluded the cases with neoadjuvant therapy, those treated or diagnosed in other centers, those diagnosed by fine-needle biopsy, Paget’s disease without invasive carcinoma component, intraductal epithelial proliferation without micro-invasion, and benign proliferation. The information was collected from the histopathological registers and medical records. All subjects were provided with informed consents for the use of biological material for scientific purposes. The study is in accordance with the principles of the 1975 Helsinki declaration, revised in 2000, and was approved by the Ethics Committee of the Hospital (#10149, approved 07.03.2014).

The information was analyzed using MedCalc Software (MedCalc Software Ltd., Ostend, Belgium) and SPSS version 24 (IBM Corp., Armonk, NY, USA). We used the Chi-square and Fisher tests to assess the relationship between quantitative variables. Correlations were calculated using Spearman’s rho coefficient. A *p*-value of less than 0.05 was considered significant.

## 3. Results

We gathered data from 235 patients with a diagnosis of breast cancer confirmed pathologically. As expected, most patients were females, respectively 97.45% (229 cases), given that breast cancer is an exceptionally rare condition in men. The median age at diagnosis was 61 years, and the mean age 59.9 ± 12.9 years. In our cohort of patients, we encountered 18 histopathological types of breast cancer (BC) with the most prevalent being invasive carcinoma of no special type (NST) and invasive lobular carcinoma (ILC). Data are presented in Table 1.

Pathologic tumor stages are summarized in Table 2.

Particularly, most men were diagnosed at an early stage, 3 (50%) with stage IA, 2 (33.33%) with stage IIA, and only one (16.67%) with stage IIIB. The mean tumor size was 2.8 ± 2.6 cm, and the median was 2.1 cm. The largest tumors were malignant phyllodes tumors, with the largest diameter of 28 cm. We found significant correlations between the size of the tumors and the presence of nodal or other localization metastases (*r* = 0.277, *p* < 0.05, respectively *r* = 0.135, *p* < 0.05). Regarding laterality, an increased frequency of breast cancer was observed on the left side. For men, the right side was affected more frequently, respectively in 4 of 6 (66.67%) patients. 

The distribution according to the differentiation grade is presented in Table 2. As expected, we found statistically significant correlations between the differentiation grade and tumor size (r = 0.335, *p* < 0.05), the presence of nodal metastases (*r* = 0.198, *p* < 0.05), or other metastases (*r* = 0.223, *p* < 0.05), and Pathological Tumor-Node-Metastasis (pTNM) classification (*r* = 0.324, *p* < 0.05). The immunohistochemical evaluation comprised of: hormonal receptors for estrogen (ER) and progesterone (PR), Human Epidermal Growth Factor Receptor 2 (HER2), Ki-67, p53, and DNA topoisomerase 2-alpha (Top2alpha). Data regarding ER, PR, HER2, and p53 are presented in Table 2. The Ki-67 marker was evaluated for 198 patients and Top2alpha for 45 patients. The mean values were 23 for Ki-67 and 25 for Top2alpha, and the medians were 18 for Ki-67 and 20 for Top2alpha.

We selected 37 (15.74%) patients with multifocal and multicentric breast carcinoma: 6 (16.22%) multicentric and 31 (83.78%) multifocal. The six multicentric carcinomas had the following histopathological types: 50% (*n* = 3) ILC, 33.33% (*n* = 2) NST, and 16.67% (*n* = 1) mixt ILC-NST. Among the patients with multifocal breast carcinoma, the prevalent histological type was NST (*n* = 27, 87.09%), followed by ILC (*n* = 2, 6.45%), CMF (*n* = 1, 3.23%), and mixt histopathological type (*n* = 1, 3.23%). Regarding the mean and median age of patients with one or multiple tumors, we did not observe significant differences. Particularly, the lowest age among multicentric breast cancer patients was 48 years, higher than that of the unicentric and multifocal tumors, respectively 26 and 24 years. The characteristics of tumors for the 37 patients with synchronous multiple breast cancer, compared to unicentric breast cancer are summarized in Table 3.

We evaluated the following parameters for all cases: index tumor, number of tumoral foci, grading of the index tumor and the tumoral foci, histological type of index tumor and of the tumoral foci, molecular subtype, and the presence of metastases (histological type, grade of differentiation, molecular type). We used the previously mentioned parameters for developing an algorithm in order to select the most aggressive tumor, namely the dominant tumor. Table 4 presents the characteristics of all 37 patients’ tumors.

In all 37 cases, the number of tumoral foci was comprised between 2 and 11. Neither contingent foci nor non-invasive ductal carcinoma were reported connecting the lesions rendering them separate foci. Needle tract implantation following initial biopsy of the tumors was unlikely. This is supported by pre-procedural mammography proof of some secondary foci, and different molecular subtypes identified between many of the foci (intertumoral heterogeneity). Almost half of the patients (45.9%, no 21–37) had more than 5 tumoral foci. The size of the index tumor varied from 10 mm to 38 mm, with a mean value of 24 mm, lower than the mean diameter of tumors from all the patients included in the current study (28 mm). The reason for this difference is that multifocal tumors have greater chances to be discovered earlier. The dimensions of secondary tumors were inconstant, although the majority had a diameter under 5 mm. In particular, tumors with 2–4 foci had a diameter similar to one of the index tumors. There were cases with similar diameters of tumoral foci (no 1, 8, 10, 34), therefore it was difficult to determine which was the index tumor. The TNM staging system would classify the tumor in example 8 from Table 4 (2 tumoral foci, 15 mm each) into the pT1 category, although it has a double tumor burden. For these particular cases, international standards have not reached a consensus. Some studies suggest summation of the tumor’s diameters or risk estimations and subsequent restaging in another prognostic class which will reflect the newly measured tumor burden [5,6]. According to the current guidelines, in cases with multiple breast carcinoma (in our study up to 11 tumors per patient), the cumulative mass is not taken into consideration for classification, hence these patients are included in a category with better prognosis and might be under-treated.

The differentiation grade of index and secondary tumors differed in 13 cases. In eight of them (no 2, 3, 9, 10, 23, 24, 25, 27), the grading of the secondary foci was higher than that of the index tumor, suggesting that the index tumor is not dominant from the perspective of aggressiveness. This finding is corroborated with cases with higher grading of nodal metastases than of the index tumor, and similar to secondary foci (no 9, 10, 25). We also encountered cases with a poorly differentiated primary tumor, and moderate to well-differentiated metastases, suggesting a paradoxical differentiation (no 19).

Regarding the tumoral histotype, most of the index and secondary tumors had similar histotypes, with 3 exceptions (8.1%). In all 3 cases (no 9, 11, 25), the index tumor was NST carcinoma, and secondary foci included ILC carcinoma; all 3 cases also had metastases: 2 cases NST (no 9, 11) and 1 case ILC (no 25). We also found mismatches regarding hormonal receptors, as follows: 11 (29.8%) cases with ER status mismatch and 12 (32.4%) cases with PR status mismatch. The HER2 status did not vary significantly in our group. The Ki-67 was the most variable parameter in our study and was discordant in 17 (45.9%) cases. The predominant molecular subtype in our population was luminal A, with 25 cases classified in this category. In 5 of these cases (no 9, 10, 12, 32, 33), we observed discordances between the index tumor and at least one secondary tumor. In secondary foci with proliferation index higher than 14% the molecular subtype changed to luminal B and despite having smaller dimensions compared to the index tumors, secondary tumors became dominant on account of their increased aggressiveness. (smaller tumor, in bold in Table 4) (no 9, 10, 12, 32, 33).

Secondary tumors also showed an extreme heterogeneity of hormonal receptors (ER and PR) but the designation of the luminal class was based on the percentage of Ki-67 expression only. One of those 5 cases (no 32) presented with a nodal metastasis with a phenotype similar to the reclassified secondary tumor (luminal B); we concluded that the secondary tumor was dominant. We also observed a case with a luminal A type index tumor, luminal B type secondary foci, and triple-negative nodal metastases (no 33).

The index tumor was luminal B type in 6 multiple breast carcinomas (no 6, 13, 14, 24, 30, 35), and none had nodal metastases; we observed discordances with secondary tumors in 3 cases (no 6, 14, 24). Only one case had overexpression of HER2 (no 26), but it did not show discrepancies between the index tumor, secondary foci, and the metastases. In addition, there were at least 4 cases where we observed mismatches between molecular patterns of breast tumors and nodal metastases: 2 cases with luminal A for breast tumors and luminal B for metastases (no 18, 28), one case with triple negative breast tumors and luminal B metastases (no 17), and one case with luminal A and B breast tumors and triple negative nodal metastases (no 33).

## 4. Discussion 

Breast cancer is a heterogeneous group of neoplasms, with significant clinical, morphological, and immunohistochemical differences within a single tumor or between tumors [11,12]. Introducing new immunohistochemical parameters allowed for better diagnosis and prognostic stratification of the tumors, with an impact on treatment management. Synchronous multiple breast cancer is the ideal prototype for studying tumoral heterogeneity, both inter- and intra-tumoral. The analysis of this subgroup was performed in order to evaluate the influence of histopathological and immunohistochemical heterogeneity on the classification of molecular subtypes, which can result in changing the therapeutic management.

The TNM staging system is considered the gold standard in classifying breast cancer. In its latest version, it does not take into consideration the multifocality and multicentricity. In cases with multiple tumoral foci, only the tumor with the highest diameter is taken into account for the pT category, while secondary tumors that are not contiguous with the primary tumor are not added to the size [4]. As a result, the cumulative mass does not impact on treatment preference. Previous studies demonstrate that synchronous multiple breast cancer has a higher risk of metastasis, of recurrence, and a worse prognosis, compared to unifocal tumors with similar staging (TNM) [6,13,14,15,16,17]. We emphasized the same observation. We noticed the presence of nodal metastases at diagnosis in 19 out of 37 patients. Their presence appeared to be associated with a higher number of invasive tumoral foci, the majority having more than four tumoral foci (index tumor included). Most of the nodal metastases had a similar histopathological and molecular subtype as the index and secondary tumors.

In our study, we encountered challenges when tumors with equal dimensions had to be classified, as the TNM and WHO staging systems recommend considering only one tumor for classification [4]. The immunohistochemical characterization of multifocal tumors demonstrates a heterogenic expression of hormonal receptors and proliferation markers. The evaluation of nodal metastases showed discordances compared to the index tumor and secondary foci. We searched for discordances between tumoral foci in our patients (*n* = 37), and we encountered the following: 3 cases (8.1%) with histopathological mismatch, 13 (35.1%) with different grades of differentiation, 11 (29.8%) with ER status mismatch, 12 (32.4%) with PR status mismatch, 8 (21.6%) with molecular phenotype mismatch, and 17 (45.9%) cases with variable Ki-67. Variability of Ki-67 is useful in determining the dominant tumor and in differentiating between luminal A and luminal B subtypes. When a luminal A index tumor is associated with a smaller focus but with a higher proliferation marker (above 14%), the secondary focus is considered luminal B and becomes the dominant tumor despite its smaller size, because it is the most aggressive of the two tumors. Aside from the numerous mismatches described above, the dominant tumor was reconsidered in 5 (13.5%) cases—one of the secondary tumors was classified as dominant, based on more aggressive histological and molecular pattern which would impact the treatment plan. However, these tumors were smaller than the index tumor, and since the TNM classification only considers one tumor (the largest one, which, as demonstrated above is not always the most aggressive one), there is a significant gap in accurately classifying, staging, and consequently establishing the prognosis and the treatment strategy for these patients.

Given the high heterogeneity between the index and the secondary tumors, we consider that more than one tumor should be evaluated in synchronous BC. Few studies treated similarly the characterization of tumoral heterogeneity. The results are presented in Table 5.

There are a few studies that support the current staging which uses only the tumor with the largest diameter for the T category. Their results showed that multiplicity had no significant influence on survival, even if it is associated with more aggressive features [16,25,26,27,28]. Although the evaluation of more tumoral foci is time-consuming and expensive, our results, as well as many other studies, suggest that it is opportune to evaluate secondary foci since the results might affect the therapeutic decision [16,19,20,22,29]. Especially the molecular heterogeneity was associated with a shorter survival, while the microscopic heterogeneity (histopathological type and grade of differentiation) did not have an influence [20]. We believe that our observations should be validated by a study on a larger lot to ensure a higher statistical power. Given the low prevalence of multiple BC and the current practice to perform molecular profiling only on the index tumor, it is difficult to enroll eligible patients in retrospective studies.

## 5. Conclusions

The latest version of the TNM staging system for breast cancer does not take into account multifocality and multiplicity. In these cases, only the tumor with the largest diameter is considered for the pT category, neglecting the importance secondary foci; therefore, the real tumor burden is underrated and the possible mismatches between the index tumor and the secondary foci, that might influence the treatment decision, are overlooked.

According to our results, the actual breast cancer staging system is perfectible, especially in the era of precision medicine. This paper highlights the importance of the total tumor burden and the molecular differences among the index tumors, secondary foci, and nodal metastases in staging and treatment of breast cancer. This is even more important in a time when personalized oncological and surgical decisions are gaining momentum. We consider that reporting the cumulative tumor burden and the most aggressive tumor could improve the accuracy of staging and improve clinical decisions.

## Figures and Tables

**Table 1:** Histopathological types of breast cancer (BC) in all patients.

Histopathological Type	Number *n* = (%)
Infiltrating ductal carcinoma NOS (NST/no special type carcinoma)	147 (62.55%)
Lobular carcinoma NOS	19 (8.09%)
Invasive carcinoma NST with medullary features (CMF)	12 (5.11%)
Carcinoma of mixed type	12 (5.11%)
Mucinous adenocarcinoma	9 (3.83%)
Malignant Phyllodes Tumor	7 (2.98%)
Metaplastic Carcinoma NOS	6 (2.55%)
Ductal Carcinoma in Situ (DCIS) with Microinvasion	6 (2.55%)
Tubular Carcinoma	2 (0.85%)
Neuroendocrine Carcinoma NOS	2 (0.85%)
Cribriform Carcinoma NOS	2 (0.85%)
Intraductal papillary adenocarcinoma with invasion	2 (0.85%)
Invasive Micropapillary Carcinoma	2 (0.85%)
Angiosarcoma	2 (0.85%)
Apocrine adenocarcinoma	1 (0.43%)
Adenoid Cystic Carcinoma	1 (0.43%)
Encapsulated papillary carcinoma with invasion	1 (0.43%)
Primary Hodgkin‘s Lymphoma of the Breast	1 (0.43%)
Primary Breast Diffuse Large B-Cell Lymphoma (DLBCL)	1 (0.43%)

NOS = not otherwise specified.

**Table 2:** Characteristics of the index tumor in all 235 patients with multiple of unifocal breast carcinoma.

Variable		No. of Patients (%)
**Gender**	Female	229 (97.45%)
	Male	6 (2.55%)
**Laterality**	Left sided	139 (59.15%)
	Right sided	78 (33.19%)
	Bilateral	4 (1.70%)
	Unknown	14 (5.96%)
**pTNM**	IA	46 (19.57%)
	IIA	61 (25.96%)
	IIB	41 (17.45%)
	IIIA	10 (4.26%)
	IIIB	19 (8.09%)
	IIIC	4 (1.7%)
	IV	33 (14.04%)
	Unknown	21 (8.94%)
**Grade of differentiation**	G1	38 (16.17%)
	G2	124 (52.77%)
	G3	59 (25.11%)
	Unknown	14 (5.96%)
**Nodal metastasis**	pN0	132 (56.17%)
	pN1	65 (17.66%)
	pN2	12 (5.11%)
	pN3	5 (2.13%)
	Unknown	21 (8.93%)
**ER status**	positive	150 (63.83%)
	negative	45 (19.15%)
	Unknown	40 (17.02%)
**PR status**	positive	153 (65.11%)
	negative	42 (17.87%)
	Unknown	40 (17.02%)
**HER2**	0	131 (55.74%)
	1+	28 (11.91%)
	2+	14 (5.96%)
	3+	13 (5.53%)
	Unknown	49 (20.85%)
**P53**	positive	26 (11.06%)
	negative	52 (22.13%)
	Unknown	157 (66.80%)

P = by histopathological examination; pTNM = Pathological Tumor-Node-Metastasis; ER = estrogen receptor, PR = progesterone receptor, HER2 = Human Epidermal Growth Factor Receptor 2.

**Table 3:** Characteristics of index tumors in patients with multifocal, multicentric, or unicentric breast cancer.

Variable		Multifocal *n* = (%)	Multicentric *n* = (%)	Unicentric *n* = (%)	*p* Value
**Age (years)**	Mean Median Minimum Maximum	60 61 24 81	60 57 48 83	60 61 26 87	NS
**Gender**	Female Male	31 (100%) 0 (0%)	6 (100%) 0 (0%)	173 (96.65%) 6 (3.35%)	0.523
**Laterality**	Left-sided Right-sided Bilateral	16 (51.61%) 14 (45.16%) 1 (3.23%)	4 (66.67%) 2 (33.33%) 0 (0%)	113 (64.57%) 59 (33.71%) 3 (1.71%)	0.728
**Grade of differentiation**	G1 G2 G3	3 (9.68%) 23 (74.19%) 5 (16.13%)	0 (0%) 4 (66.67%) 2 (33.33%)	30 (18.07%) 92 (55.42%) 44 (26.51%)	0.605
**Nodal metastasis**	pN0 pN1 pN2 pN3	18 (58.06%) 12 (38.71%) 1 (3.23%) 0 (0%)	4 (66.67%) 1 (16.67%) 1 (16.67%) 0 (0%)	108 (61.71%) 52 (29.71%) 10 (5.71%) 5 (2.86%)	0.698
**Molecular subtype**	Luminal A Luminal B Triple negative HER2+	19 (61.29%) 6 (19.35%) 5 (16.13%) 1 (3.23%)	6 (100%) 0 (0%) 0 (0%) 0 (0%)	70 (46.67%) 51 (34%) 18 (12%) 11 (7.33%)	0.266

P = by histopathological examination; ER = estrogen receptor, PR = progesterone receptor, HER2 = Human Epidermal Growth Factor Receptor 2; IHC = immunohistochemical.

**Table 4:** Evaluation of intertumoral heterogeneity in multiple breast carcinoma.

No.	No of foci	Size (mm)	Grade of Differentiation	Histotype	Molecular Subtype	Metastases		
						Grade	Histotype	Molecular Subtype
**1**	**2**	**10/10**	**1/1**	**D/D**	LuA/LuA			
**2**	2	**12**/5	1/2	D/D	LuA/LuA			
**3**	2	**11**/3	1/2	D/D	LuA/LuA			
**4**	2	**11**/4	2/2	D/D	LuA/LuA			
**5**	2	**12**/5	2/2	D/D	LuA/LuA			
**6**	2	**12**/10	2/2	D/D	LuB/LuA			
**7**	2	**12**/11	2/2	D/D	TN/TN			
**8**	2	**15/15**	2/2	MX	LuA/LuA			
**9**	2	16/**12**	2/3	D/L	LuA/LuB	3	D	LuA
**10**	2	18/**18**	2/3	D/D	LuA/LuB	3	D	
**11**	3	**20**/10/3	2/2/2	D/D/L	LuA/LuA/LuA	2	D	LuA
**12**	3	20/**11**/3	2/2/2	D/D/D	LuA/LuB/LuA			
**13**	3	**20**/11/2	2/2/2	D/D/D	LuB/LuB/LuB			
**14**	3	**21**/5/5	2/2/2	D/D/D	LuB/LuA/LuA			
**15**	3	**26**/4/3	3/3/3	D/D/D	LuA/LuA/LuA	3	D	LuA
**16**	4	**17**/5/5/3	2/2/2/2	D/D/D…	LuA/LuA/LuA…			
**17**	4	**18**/6/6/4	2/2/2/2	D/D/D…	TN/TN/TN…	3	D	LuB
**18**	4	**23**/11/5/5	2/2/2/2	MX	LuA/LuA/LuA…	2	D	LuB
**19**	4	**26**/4/4/2	3/3/3/3	D/D/D…	LuA/LuA/LuA…	2	D	LuA
**20**	5	**36**/12/4/4/3	3/3/3/3/3	M/M/M…	TN/TN/TN…	3	M	TN
**21**	6	**22**/11/6/6/4/1	2/2/2/2/2/2	L/L/L	LuA/LuA/LuA…			
**22**	7	**20**/11/7/4/4/2/1	2/2/2/2/2/2/2	D/D/D…	LuA/LuA/LuA…	2	D	LuA
**23**	7	**20**/12/7/4/5/2/1	2/3/2/2/2/2/1	D/D/D…	LuA/LuA/LuA…			
**24**	7	**21**/11/7/4/4/2/1	2/3/3/2/2/2/2	D/D/D…	LuB/LuA/LuA…			
**25**	7	**26**/18/7/4/4/2/1	2/3/3/3/3/2/2	D/L/L	LuA/LuA/LuA…	3	L	LuA
**26**	8	**18**/10/3/2/2/1/1/1	2/2/2/2/2/2/2/2	L/L/L	HER/HER/HER…	2	L	HER
**27**	8	**23**/11/5/4/2/1/1/1	2/2/2/3/2/1/1/1	D/D/D…	LuA/LuA/LuA…			
**28**	8	**28**/12/4/3/3/1/1/1	2/2/2/2/1/1/1/1	D/D/D…	LuA/LuA/LuA…	2	D	LuB
**29**	8	**30**/10/3/2/2/1/1/1	3/3/3/3/3/3/3/3	D/D/D…	TN/TN/TN…	3	D	TN
**30**	9	**25**/14/3/2/1/1/1/1…	2/2/2/2/1/1/1/1/1	D/D/D…	LuB/LuB/LuB…			
**31**	9	**38**/10/6/2/2/1/1/1…	3/3/3/3/3/3/3/3/3	L/L/L…	LuA/LuA/LuA…	3	L	LuA
**32**	10	26/**15**/4/4/2/2/1/1…	2/2/2/1/1/1/1/1/1/1	D/D/D…	LuA/LuB/LuA…	2	D	LuB
**33**	10	27/**10**/5/5/2/1/1/1…	2/2/2/2/2/2/2/2/2/2	D/D/D…	LuA/LuB/LuA…	3	D	TN
**34**	11	**10**/10/5/2/2/1/1/1…	3/2/2/2/2/2/2/2/2/2/2	D/D/D…	TN/TN/TN…	3	D	TN
**35**	11	**18**/12/3/3/2/1/1/1…	2/2/2/2/2/2/2/2/2/2/2	D/D/D…	LuB/LuB/LuB…			
**36**	11	**32**/18/4/4/2/1/1/1…	2/2/2/2/2/2/2/2/2/2/2	L/L/L…	LuA/LuA/LuA…	2	L	LuA
**37**	11	**35**/10/3/2/2/1/1/1…	3/3/3/3/3/3/3/3/3/3/3	L/L/L…	LuA/LuA/LuA…	3	L	LuA

D = Ductal Carcinoma (NST); L = Invasive Lobular Carcinoma; MX = Mixt Carcinoma; M = Carcinoma with Medullary Characteristics; LuA = Luminal A; LuB = Luminal B; TN = Triple Negative; HER = HER2-enriched. Highlighted rows—multicentric carcinoma; non-highlighted rows—multifocal carcinoma; Numbers in bold—the dominant tumor.

**Table 5:** Tumoral heterogeneity in other studies.

Study	Year	Number of Patients	Mismatch in	N = (%)
**Lang et al. [18]**	2017	156	ER PR Grading Ki-67 HER2	6 (3.85%) 4 (2.56%) 6 (3.85%) 9 (5.77%) 2 (1.28%)
**Baretta et al. [19]**	2015	4403	ERP R	422 (10%) 816 (19%)
Pekar et al. [20] *	2014	110	Histologic type Grading Molecular subtype	16 (14.5%) 6 (5.5%) 11–14 (10–12.7%)
Buggi et al. [21] **	2012	113	ER PR Grading Ki-67 HER2	5 (4.4%) 18 (15.9%) 21 (18.6%) 17 (15%) 11 (9.7%)
**Choi et al. [22]**	2012	65	ER PR HER2 Molecular subtypes	2 (3%) 7 (11%) 4 (6%) 5 (8%)
**Boros et al. [23]**	2008	91	Histological type Grading	11 (12.08%) 9 (9.89%)
**Garimella et al. [24]**	2007	18	ER PR	0 (0%) 2 (11%)

* The authors also reported that if phenotyping of the secondary foci was performed before therapy, the results would have influenced the therapy choice in eight patients. ** In this study, eleven patients received different adjuvant therapy than what would have been received if only the primary tumor was assessed. ER = estrogen receptor, PR = progesterone receptor; HER2 = Human Epidermal Growth Factor Receptor 2.

## References

[1] Yeo S.K., Guan J.L. (2017). Breast Cancer: Multiple Subtypes within a Tumor?. Trends Cancer.

[2] Chu J., Bae H., Seo Y., Cho S.Y., Kim H.-S., Cho E.Y. (2018). The Prognostic Impact of Synchronous Ipsilateral Multiple Breast Cancer: Survival Outcomes according to the Eighth American Joint Committee on Cancer Staging and Molecular Subtype. J. Pathol. Transl. Med..

[3] Winters Z.E., Horsnell J., Elvers K.T., Maxwell A.J., Jones L.J., Shaaban A.M., Schmid P., Williams N.R., Beswick A., Greenwood R. (2018). Systematic review of the impact of breast-conserving surgery on cancer outcomes of multiple ipsilateral breast cancers. BJS Open.

[4] Amin M.B., Edge S.B., Greene F.L., Byrd D.R., Brookland R.K., Washington M.K., Gershenwald J.E., Compton C.C., Hess K.R., Sullivan D.C., AJCC (American Joint Committee on Cancer) (2018). Cancer Staging Manual.

[5] Kelemen G., Farkaš V., Debrah J., Ormandi K., Voros A., Kaizer L., Varga Z., Lazar G., Kahán Z. (2012). The relationship of multifocality and tumor burden with various tumor characteristics and survival in early breast cancer. Neoplasma.

[6] Coombs N.J., Boyages J. (2005). Multifocal and Multicentric Breast Cancer: Does Each Focus Matter?. J. Clin. Oncol..

[7] Weissenbacher T., Zschage M., Janni W., Jeschke U., Dimpfl T., Mayr R., Rack B., Schindlbeck C., Friese K., Dian D. (2010). Multicentric and multifocal versus unifocal breast cancer: Is the tumor-node-metastasis classification justified?. Breast Cancer Res. Treat..

[8] Neri A., Marrelli D., Megha T., Bettarini F., Tacchini D., De Franco L., Roviello F. (2015). Clinical significance of multifocal and multicentric breast cancers and choice of surgical treatment: A retrospective study on a series of 1158 cases. BMC Surg..

[9] Lynch S.P., Lei X., Hsu L., Meric-Bernstam F., Buchholz T.A., Zhang H., Hortobágyi G.N., Gonzalez-Angulo A.M., Valero V. (2013). Breast cancer multifocality and multicentricity and locoregional recurrence. Oncologist.

[10] Gradinaru S.E., Bumbea H., Onisai M.C., Stoicea M. (2017). Neuroendocrine differentiation in invasive lobular breast carcinoma. Acta Endocrinol. (Buchar)..

[11] Polyak K. (2011). Heterogeneity in breast cancer. J. Clin. Invest..

[12] Aleskandarany M.A., Vandenberghe M.E., Marchiò C., Ellis I.O., Sapino A., Rakha E.A. (2018). Tumor Heterogeneity of Breast Cancer: From Morphology to Personalised Medicine. Pathobiology.

[13] Andea A.A., Wallis T., Newman L.A., Bouwman D., Dey J., Visscher D.W. (2002). Pathologic analysis of tumor size and lymph node status in multifocal/multicentric breast carcinoma. Cancer.

[14] Fish E.B., Chapman J.A., Link M.A. (1998). Assessment of tumor size for multifocal primary breast cancer. Ann. Surg. Oncol..

[15] Egan R.L. (1982). Multicentric breast carcinomas: Clinical-radiographic-pathologic whole organ studies and 10-year survival. Cancer.

[16] Panuta A., Radu I., Gafton B. (2019). Multiple versus unifocal breast cancer: Clinocopathological and immunohistochemical difference. Rom. J. Morphol. Embryol..

[17] Paredes-Aracil E., Palazón-Bru A., La Rosa D.M.F.-D., Ots-Gutiérrez J.R., Rosique A.F.C., Gil-Guillén V.F. (2017). A scoring system to predict breast cancer mortality at 5 and 10 years. Sci. Rep..

[18] Lang Z., Wu Y., Li C., Wang X., Qu G. (2017). Multifocal and Multicentric Breast Carcinoma: A Significantly More Aggressive Tumor than Unifocal Breast Cancer. Anticancer Res..

[19] Baretta Z., Olopada O.I., Huo D. (2015). Heterogeneity in Hormone-Receptor Status and Survival Outcomes among Women with Synchronous and Metachronous Bilateral Breast Cancers. Breast.

[20] Pekar G., Gere M., Tarján M., Hellberg D., Tot T. (2014). Molecular phenotype of the foci in multifocal invasive breast carcinomas: Intertumoral heterogeneity is related to shorter survival and may influence the choice of therapy. Cancer.

[21] Buggi F., Folli S., Curcio A., Casadei-Giunchi D., Rocca A., Pietri E., Medri L., Serra L. (2012). Multicentric/multifocal breast cancer with a single histotype: Is the biological characterization of all individual foci justified?. Ann. Oncol..

[22] Choi Y., Kim E.J., Seol H., Lee H.E., La Yun B., Kim S.M., Kim J.H., Kim S.-W., Choe G., Park S.Y. (2012). The hormone receptor, human epidermal growth factor receptor 2, and molecular subtype status of individual tumor foci in multifocal/ multicentric invasive ductal carcinoma of breast. Hum. Pathol..

[23] Boros M., Marian C., Moldovan C., Stolnicu S. (2012). Morphological heterogeneity of the simultaneous ipsilateral invasive tumor foci in breast carcinoma: A retrospective study of 418 cases of carcinomas. Pathol. Res. Pract..

[24] Garimella V., Long E.D., O’Kane S.L., Drew P.J., Cawkwell L. (2007). Oestrogen and progesterone status of individual foci in multifocal invasive ductal cancer. Acta Oncol..

[25] Lynch S.P., Lei X., Chavez-Macgregor M., Hsu L., Meric-Bernstam F., Buchholz T.A., Zhang A., Hortobagyi G.N., Valero V., Gonzalez-Angulo A.M. (2012). Multifocality and multicentricity in breast cancer and survival outcomes. Ann. Oncol..

[26] Pedersen L., Gunnarsdottir K., Rasmussen B., Moeller S., Lanng C. (2004). The prognostic influence of multifocality in breast cancer patients. Breast.

[27] Vlastos G., Rubio I.T., Mirza N.Q., Newman L.A., Aurora R., Alderfer J., Buzdar A.U., Singletary S.E. (2000). Impact of multicentricity on clinical outcome in patients with T1-2, N0-1, M0 breast cancer. Ann. Surg. Oncol..

[28] Djordjevic-Jovanovic L., Karanikolic A., Bojic T., Pesic I., Djordjevic M., Marinkovic M. (2017). Characteristics and outcomes of patients with multifocal/multicentric and unifocal breast cancer. J. BUON.

[29] Boros M., Podoleanu C., Georgescu R., Moldovan C., Molnar C., Stolnicu S. (2015). Multifocal/multicentric breast carcinomas swoing intertumoral heterogeneity: A comparison of histological tumor type and Nottingham histological grade of primary tumor and lymph node metastasis. Pol. J. Pathol..

